# HCV core protein inhibits polarization and activity of both M1 and M2 macrophages through the TLR2 signaling pathway

**DOI:** 10.1038/srep36160

**Published:** 2016-10-27

**Authors:** Qianqian Zhang, Yang Wang, Naicui Zhai, Hongxiao Song, Haijun Li, Yang Yang, Tianyang Li, Xiaolin Guo, Baorong Chi, Junqi Niu, Ian Nicholas Crispe, Lishan Su, Zhengkun Tu

**Affiliations:** 1 Institute of Translational Medicine, the First Hospital of Jilin University, Changchun, China; 2 Department of Hepatobiliary and Pancreatic Diseases, the First Hospital of Jilin University, Changchun, China; 3 College of Clinical Medicine, Jining Medical University, Jining, China; 4 Department of Pathology, University of Washington, Seattle, WA, USA; 5 Lineberger Comprehensive Cancer Center, School of Medicine, University of North Carolina at Chapel Hill, Chapel Hill, NC 27599, USA

## Abstract

Hepatitis C virus (HCV) establishes persistent infection in most infected patients, and eventually causes chronic hepatitis, cirrhosis, and hepatocellular carcinoma in some patients. Monocytes and macrophages provide the first line of defense against pathogens, but their roles in HCV infection remains unclear. We have reported that HCV core protein (HCVc) manipulates human blood-derived dendritic cell development. In the present study, we tested whether HCVc affects human blood-derived monocyte differentiating into macrophages. Results showed that HCVc inhibits monocyte differentiation to either M1 or M2 macrophages through TLR2, associated with impaired STATs signaling pathway. Moreover, HCVc inhibits phagocytosis activity of M1 and M2 macrophages, M1 macrophage-induced autologous and allogeneic CD4^+^ T cell activation, but promotes M2 macrophage-induced autologous and allogeneic CD4^+^ T cell activation. In conclusion, HCVc inhibits monocyte-derived macrophage polarization via TLR2 signaling, leading to dysfunctions of both M1 and M2 macrophages in chronic HCV infected patients. This may contribute to the mechanism of HCV persistent infection, and suggest that blockade of HCVc might be a novel therapeutic approach to treating HCV infection.

Infection with hepatitis C virus (HCV) results in persistent liver disease in the majority of infected individuals, and HCV-associated end-stage liver disease is now the leading indication for liver transplantation in the world^1^,^2^. The ability of HCV to establish persistent infection with great success in human has been attributed, in part, to a variety of strategies to evade host immune and IFN-induced defenses^3^. Epidemiological studies suggest that up to 20% of acutely infected HCV patients can resolve the infection without treatment, which implies that innate and/or adaptive immune responses are indeed capable of controlling the outcome of HCV infection^4^,^5^. Many studies have highlighted the importance of the T cell response for viral clearance and attributed persistent infection to an insufficient T cell response, but HCV interferes with the activation of the T cell response through innate immune cells^6^,^7^,^8^. Chronic HCV infection is associated with the activation of inflammatory cells and cytokines cascade, including monocytes or macrophages activation and recruitment.

Macrophages differentiate from peripheral monocytes, and are present as phagocytic cells in all tissues. Kupffer cells are the liver resident macrophages, consisting as much as 25% of the cells in the liver^9^. Monocytes/macrophages play an important role in immune surveillance and immunoregulation depending on their functions of phagocytosis and antigen presentation^10^,^11^. Peripheral monocytes tend to differentiate into different subtypes of macrophages depending on the tissue microenvironment. The Th1 cytokine IFN-γ and the ligand of TLR4, lipopolysaccharide (LPS), polarize monocytes towards classically activated (M1) macrophages, which produce pro-inflammatory cytokines, such as TNF-α, IL-12, subsequently facilitating clearance of pathogens and resulting in tissue damage. In contrast, exposure to Th2 cytokines as IL-4 and IL-13, monocytes differentiate to alternatively activated macrophages (M2 macrophages) with the production of anti-inflammatory mediators IL-10, which act the role of anti-inflammation and wound healing^12^,^13^. More recently, some studies have reported that HCV induces monocyte differentiation and polarization of macrophages that promote liver fibrogenesis in chronic infection^14^. Other studies have shown that HCV infection dampens M1 macrophage polarization *ex vivo* and *in vitro*^15^. However, how monocyte differentiation and macrophage polarization are affected in HCV infection remains incompletely understood.

HCV core protein (HCVc) is an RNA-binding protein that is highly conserved and has a basic N-terminal region and a hydrophobic C-terminus^16^, participating in the formation of the viral nucleocapsid and modulating the host immune responses^12^. HCVc induces TLR2-mediated activation of monocytes and macrophages to result in the activation of the inflammatory cascade, including the activation of IRAK-1 kinase, NF-κB, MAPK, and TNF-α production^17^,^18^,^19^. Increased level of TLR2 and TLR4 expression were observed in peripheral monocytes of patients with HCV infection, and increased expression of TLR2 is particularly associated with an increase in circulating TNF-α level and hepatic inflammatory activity^20^. Our previous study showed that HCVc inhibits monocyte differentiation into dendritic cells (DCs)^21^.

In the current study, we tested the hypothesis that HCV affects monocyte-derived macrophages. Our data showed that HCV infection suppresses monocyte differentiation into both M1 and M2 macrophages through a TLR2/STATs signaling pathway. Moreover, HCVc inhibits phagocytosis by both M1 and M2 macrophages, M1 macrophage-induced autologous and allogeneic CD4^+^ T cell proliferation, but promotes M2 macrophage-induced autologous and allogeneic CD4^+^ T cell proliferation.

## Results

### HCV infection suppresses monocytes differentiation to macrophages

**Figure 1:**
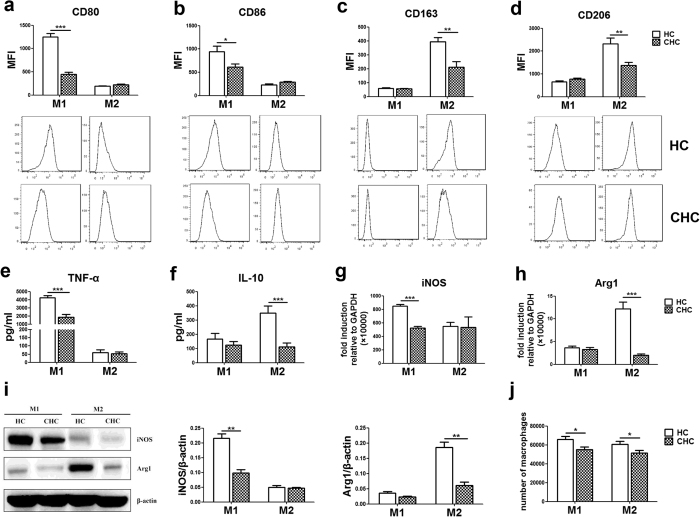
Human peripheral monocytes differentiation into M1 or M2 macrophages are impaired in chronic HCV infected patients. Purified monocytes from 17 chronic HCV patients (CHC) and 17 healthy controls (HC) were polarized to M1 and M2 macrophages. (**a–d**) The expression level of CD80 and CD86 on M1 macrophages, CD163 and CD206 on M2 macrophages in chronic HCV infected patients are significantly lower than healthy controls. (**e,f**) TNF-α production of M1 macrophages and IL-10 production of M2 macrophages in chronic HCV infected patients are significantly lower than healthy controls. (**g,h**) The mRNA expression level of iNOS by M1 macrophages and Arg1 by M2 macrophages in chronic HCV infected patients are significantly lower than healthy controls. (**i**) The protein level of iNOS of M1 macrophages and Arg1 of M2 macrophages in chronic HCV infected patients are significantly lower than healthy controls. (**j**) The total number of macrophage is significantly reduced in CHC patients after polarization. (n = 17, *P < 0.05, **P < 0.01, ***P < 0.001).

To test our hypothesis that HCV affects monocyte-derived macrophage polarization, 17 chronic HCV patients (CHC) and 17 healthy controls (HC) were enrolled in the study (Table 1). Purified monocytes from healthy donors and patients with HCV infection were polarized to M1 and M2 macrophages. The expression of phenotypic surface markers were analyzed by flow cytometry. Production of the cytokines TNF-α and IL-10, reflective of the pro-inflammatory and anti-inflammatory function of M1 and M2 macrophages respectively, were assessed using ELISA. The mRNA and proteins expression levels of iNOS (a marker of M1) and Arg1 (a marker of M2) were detected by RT-qPCR and western blot. As expected, the expression levels of CD80 (1248.0 ± 79.3 Vs 451.1 ± 41.9, *P* < 0.0001, Fig. 1a) and CD86 (943.2 ± 122.2 Vs 614.6 ± 62.0, *P* = 0.0225, Fig. 1b) of M1 macrophages, CD163 (1248.0 ± 79.3 Vs 451.1 ± 41.9, *P* = 0.0014, Fig. 1c) and CD206 (2315.0 ± 261.8 Vs 1376.0 ± 140.7, *P* = 0.0034, Fig. 1d) of M2 macrophage from HCV infected patients were significantly lower than that of healthy controls. TNF-α release (4243.0 ± 270.7 Vs 1888.0 ± 344.5, *P* < 0.001, Fig. 1e) and iNOS expression (mRNA: 848.1 ± 26.2 Vs 522.2 ± 27.5, *P* < 0.001, Fig. 1g; protein: 0.22 ± 0.01 Vs 0.10 ± 0.01, *P* = 0.0029, Fig. 1i) of M1 macrophages, IL-10 release (349.4 ± 50.6 Vs 111.9 ± 27.3, *P* < 0.001, Fig. 1f) and Arg1 expression (mRNA: 12.2 ± 1.5 Vs 2.0 ± 0.4, *P* < 0.001, Fig. 1h; protein: 0.19 ± 0.02 Vs 0.06 ± 0.01, *P* = 0.0039, Fig. 1i) of M2 macrophages from HCV infected patients are significantly lower than that of healthy individuals as well. Moreover, the total numbers of both M1 (65760 ± 3455 Vs 55060 ± 2703, *P* = 0.0204) and M2 (60710 ± 3002 Vs 51410 ± 2912, *P* = 0.0335, Fig. 1j) macrophages are significantly reduced in CHC patients after polarization, compared with healthy donors. Also, we have performed the viability and phenotypical characterization of monocytes between HCV patients and healthy donors baseline, and no significant differences were observed between the two groups (Supplement Fig. S1b,c). To exclude any impact of sex differences, we analyzed the data to compare the effect of sex differences on macrophage differentiation/polarization, and found no significant difference. These results suggest that human peripheral monocytes from chronic HCV infected patients fail to polarize toward M1 and M2 macrophages as strongly as cells from healthy donors.

**Figure 2:**
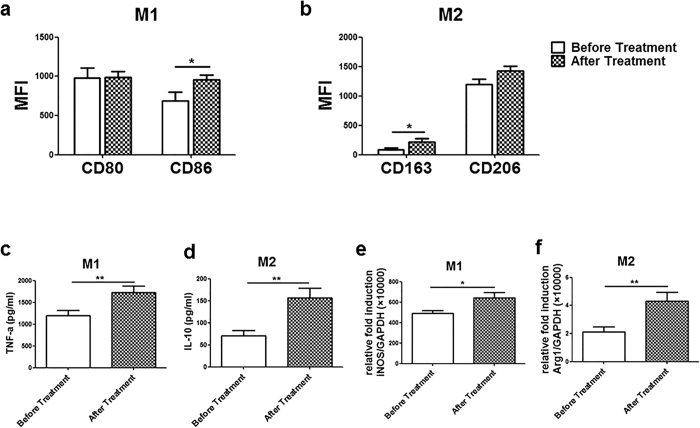
Direct-acting antivirals (DAAs) partially rescues the differentiation of macrophages. Eight HCV infected patients administrated with DAAs (NS5A inhibitor) were enrolled. Venous bloods were collected before and after treatment. Purified monocytes were polarized to M1 and M2 macrophages. (**a,b**) The expression level of CD86 on M1 macrophages, and CD163 on M2 macrophages after treatment are significantly higher than before treatment. (**c,d**) TNF-α production of M1 macrophages, and IL-10 production of M2 macrophages after treatment are significantly higher than before treatment. (**e,f**) The mRNA expression level of iNOS by M1 macrophages and Arg1 by M2 macrophages after treatment are significantly higher than before treatment. (n = 8, *P < 0.05, **P < 0.01, ***P < 0.001).

A remarkable evolution in HCV treatment with direct-acting antivirals (DAAs) drugs emerges, which has resulted in higher viral clearance rate^22^. We next investigated whether HCV reduction or eradication by DAAs therapy lead to the restoration of macrophages polarization. Eight HCV infected patients administrated with a NS5A inhibitor were enrolled in the studies (Table 2). Venous blood was withdrawn before therapy and 7 days after the treatment. Purified monocytes were polarized to M1 and M2 macrophages as mentioned above. The results were shown that the expression of CD86 (690.4 ± 107.4 Vs 955.5 ± 61.4, *P* = 0.0317, Fig. 2a) on M1 macrophages and CD163 (88.6 ± 28.0 Vs 221.5 ± 53.4, *P* = 0.0446, Fig. 2b) on M2 macrophages after treatment were up-regulated significantly, compared with before treatment. TNF-α production (1197.0 ± 117.0 Vs 1727.0 ± 145.2, *P* = 0.008, Fig. 2c) and iNOS expression (492.4 ± 25.8 Vs 643.3 ± 53.3, *P* = 0.0214, Fig. 2e) of M1 macrophages, IL-10 production (70.8 ± 11.8 Vs 156.3 ± 22.0, *P* = 0.0018, Fig. 2d) and Arg1 expression (2.1 ± 0.4 Vs 4.3 ± 0.6, *P* = 0.0068, Fig. 2f) of M2 macrophages after treatment were up-regulated significantly, compared with before treatment. As a control, purified monocytes from healthy donors were polarized to M1 and M2 macrophages in the presence of NS5A inhibitor, and their differentiation was found not to be suppressed (Supplementary Fig. S2a–d). These results suggest that reduction or eradication of viruses by the NS5A inhibitor led to relief of the inhibition of macrophage polarization.

### HCVc suppresses macrophage polarization

It has been reported that HCVc induces TLR2-mediated activation of monocytes^17^,^19^,^23^, and manipulates monocyte differentiation into dendritic cells^21^. Thus, we next investigated whether the impaired M1 and M2 macrophage polarization in patients with HCV infection was mediated by HCVc. Purified monocytes from healthy donors were differentiated into M1 and M2 macrophages in presence or absence of HCVc.

**Figure 3:**
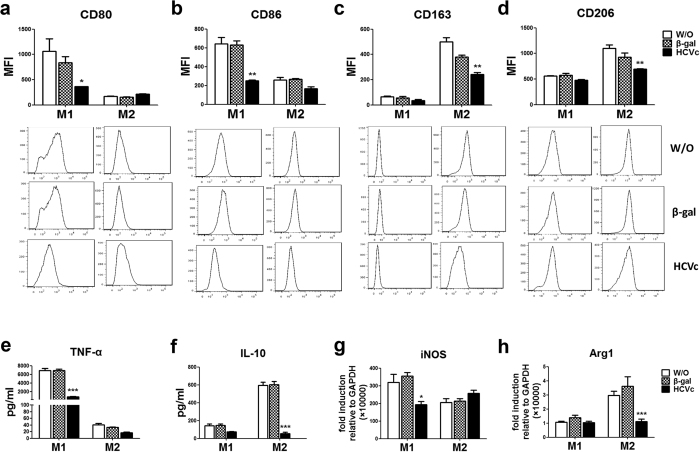
HCVc inhibits monocyte differentiation to both M1 and M2 macrophage. Purified monocytes from health individuals were polarized to M1 and M2 macrophages in the presence or absence of HCV core protein (HCVc) and β-galactosidase (β-gal, negative control). (**a–d**) HCVc inhibits the expression level of CD80 and CD86 on M1 macrophages, CD163 and CD206 on M2 macrophages, compared with β-gal and medium. (**e,f**) HCVc inhibits TNF-α production of M1 macrophages, and IL-10 production of M2 macrophages. (**g,h**) HCVc inhibits the mRNA expression level of iNOS by M1 macrophages, and Arg1 by M2 macrophages. (n = 5, *P < 0.05, **P < 0.01, ***P < 0.001).

Optimal HCVc concentration was determined as described previous^21^, and 10 μg/mL was used as the standard concentration in most experiments (Supplementary Fig. S2e). As expected, HCVc down-regulated the expression of CD80 (1066.0 ± 247.0 Vs 364.0 ± 2.0, *P* = 0.0468, Fig. 3a) and CD86 (640.7 ± 68.2 Vs 249.7 ± 7.4, *P* = 0.0047, Fig. 3b) on M1 macrophages, as well as TNF-α production (6869.0 ± 552.2 Vs 873.5 ± 51.0, *P* < 0.0001, Fig. 3e), and iNOS mRNA expression level (319.7 ± 45.3 Vs 192.9 ± 18.5, *P* = 0.0269, Fig. 3g), compared with the medium and β-gal controls. Similarly, HCVc also down-regulated the expression of CD163 (499.0 ± 35.0 Vs 243.0 ± 14.2, *P* = 0.0025, Fig. 3c) and CD206 (1098.0 ± 73.4 Vs 697.7 ± 5.2, *P* = 0.0056, Fig. 3d) on M2 macrophages, as well as IL-10 production (596.5 ± 35.1 Vs 54.8 ± 15.5, *P* < 0.0001, Fig. 3f), and Arg-1 mRNA expression level (3.0 ± 0.3 Vs 1.1 ± 0.2, *P* = 0.0004, Fig. 3h), compared with the medium and β-gal controls. These results indicate that HCVc suppressed both M1 and M2 macrophage polarization, which is consistent with chronic HCV infection.

### HCVc inhibits macrophages polarization through TLR2/STATs signaling pathway

**Figure 4:**
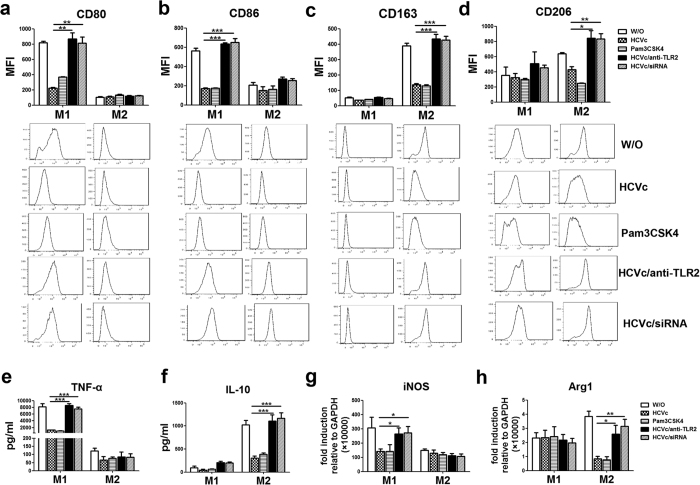
HCVc inhibits macrophage polarization through engagement with TLR2. Purified monocytes from healthy individuals were polarized to M1 and M2 macrophages in the presence or absence of HCVc and Pam3CSK4, and purified monocytes pre-treated with anti-TLR2 antibody or transfected with siRNAs targeting TLR2 were polarized to M1 and M2 macrophages in the presence of HCVc. Pam3CSK4 as well as HCVc inhibits the expression of CD80 (**a**) and CD86 (**b**), TNF-α production (**e**), and mRNA expression level of iNOS (**g**) by M1 macrophages, the expression of CD163 (**c**) and CD206 (d), IL-10 production (**f**), and mRNA expression level of Arg-1 (h) byM2 macrophages. (**a–h**) The inhibitions of HCVc-induced macrophages polarization were restored by anti-TLR2 antibody and TLR2-siRNAs. (*P < 0.05, **P < 0.01, ***P < 0.001).

We further investigated whether the inhibition of macrophages polarization by HCVc is mediated through engagement with TLR2. Purified monocytes were pretreated with anti-TLR2 antibody and TLR2 siRNA, and then differentiated into M1 and M2 macrophages in the presence of HCVc. Pam3CSK4, a classic TLR2 ligand was used as control for HCVc engagement with TLR2. The results are shown as Fig. 4. Blockade of HCVc/TLR2 engagement with anti-TLR2 antibody and TLR2 siRNA up-regulated the expression of CD80 (anti-TLR2 antibody: 223.0 ± 11.5 Vs 870.7 ± 75.9, *P* = 0.0011; TLR2 siRNA: 223.0 ± 11.5 Vs 813.3 ± 78.4, *P* = 0.0017, Fig. 4a) and CD86 (anti-TLR2 antibody: 169.0 ± 8.8 Vs 633.0 ± 13.5, *P* < 0.0001; TLR2 siRNA: 169.0 ± 8.7 Vs 651.7 ± 37.3, *P* = 0.0002, Fig. 4b) on M1 macrophages, as well as TNF-α production (anti-TLR2 antibody: 1320 ± 52.5 Vs 8603 ± 582.7, *P* < 0.0001; TLR2 siRNA: 1320 ± 52.5 Vs 7617 ± 443.6, *P* < 0.0001, Fig. 4e), and iNOS mRNA expression level (anti-TLR2 antibody: 140.8 ± 18.4 Vs 263.5 ± 44.1, *P* = 0.0279; TLR2 siRNA: 140.8 ± 18.4 Vs 271.5 ± 45.6, *P* = 0.0241, Fig. 4g), compared with HCVc. Likewise, blockade of HCVc/TLR2 engagement with anti-TLR2 antibody and TLR2 siRNA also up-regulated the expression of CD163 (anti-TLR2 antibody: 137.7 ± 5.4 Vs 435.3 ± 28.2, *P* = 0.0005; TLR2 siRNA: 137.7 ± 5.4 Vs 426.8 ± 26.1, *P* = 0.0004, Fig. 4c) and CD206 (anti-TLR2 antibody: 428.0 ± 40.1 Vs 843.0 ± 102.8, *P* = 0.0198; TLR2 siRNA: 428.0 ± 40.1 Vs 832.0 ± 73.2, *P* = 0.0084, Fig. 4d) on M2 macrophages, as well as IL-10 production (anti-TLR2 antibody: 303.7 ± 43.9 Vs 1104 ± 138.4, *P* < 0.0001; TLR2 siRNA: 303.7 ± 43.9 Vs 1166 ± 123.2, *P* < 0.0001, Fig. 4f), and Arg-1 mRNA expression level (anti-TLR2 antibody: 0.9 ± 0.2 Vs 2.6 ± 0.6, *P* = 0.0258; TLR2 siRNA: 0.9 ± 0.2 Vs 3.2 ± 0.5, *P* = 0.0012, Fig. 4h), compared with HCVc. In addition, Pam3CSK4 suppresses M1 and M2 macrophages polarization as HCVc does.

**Figure 5:**
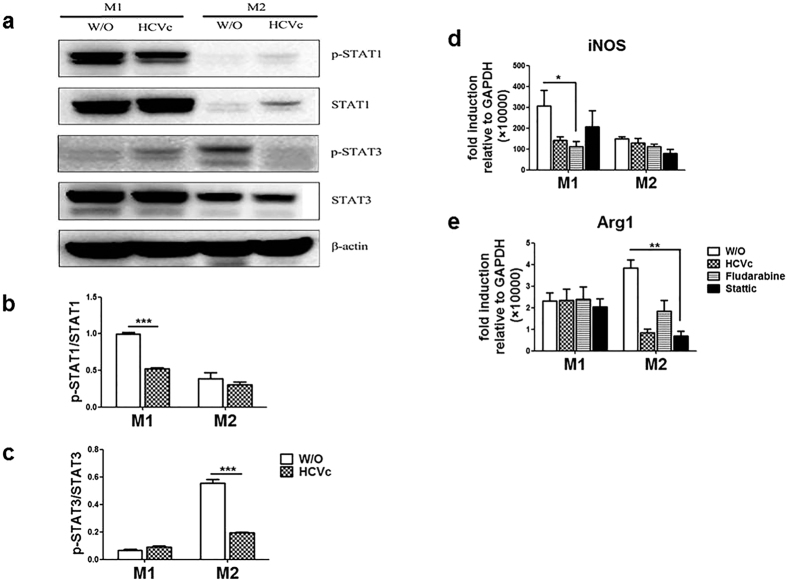
HCVc inhibits macrophage polarization correlated with inhibiton of STAT1/STAT3 activation. (**a**) Purified monocytes from healthy individuals were polarized to M1 and M2 macrophages in the presence or absence of HCVc, pSTAT1, pSTAT1, total STAT1 and STAT3 were detected by western blot. Shown is one of three representative experiments. (**b,c**) Statistical analysis of three experiments of western blot. (**d,e**) The inhibitors of STAT1 (Fludarabine) and STAT3 (Stattic) inhibit the mRNA expression levels of iNOS and Arg1 in M1 and M2 macrophages, respectively. (*P < 0.05, **P < 0.01, ***P < 0.001).

The network of molecular mediators that regulate M1 and M2 polarization is not fully understood. It has been reported that IFN-γ binding to its receptor on cell surface leads to the activation of receptor-associated JAKs, which in turn causes STAT1 to initiate transcription of genes that promote M1 macrophages-associated functions^24^,^25^. In contrast, IL-4 and IL-13 promote the polarization of M2 macrophages by activation of STAT3^26^. In order to further explore the mechanism of HCVc-manipulated monocyte differentiation into macrophages, STAT1 phosphorylation in M1 macrophages and STAT3 phosphorylation in M2 macrophages were detected by western blot. As shown in Fig. 5, the phosphorylation of STAT1 (0.10 ± 0.02 Vs 0.52 ± 0.02, *P* < 0.0001) in HCVc-treated M1 macrophages and the phosphorylation of STAT3 (0.55 ± 0.03 Vs 0.19 ± 0.01, *P* < 0.0001) in HCVc-treated M2 macrophages were hampered significantly, compared with controls. The inhibitors of STAT1 (Fludarabine) and STAT3 (Stattic) were added into M1 and M2 macrophage polarization experiments respectively. As shown in Fig. 5d,e, Fludarabine inhibited iNOS expression level of M1 macrophages (307.8 ± 75.1 Vs 110.8 ± 25.4, *P* = 0.0321), and Stattic inhibited Arg1 expression level of M2 macrophages (3.9 ± 0.4 Vs 1.8 ± 0.5, *P* = 0.0091).

Take together, We conclude that HCVc inhibits macrophages polarization through TLR2/STATs signaling pathway, and HCVc does not selectively impact M1 or M2 macrophages. Instead, it inhibits the activation of both macrophage subsets.

### HCVc inhibits the functions of M1 and M2 macrophages

**Figure 6:**
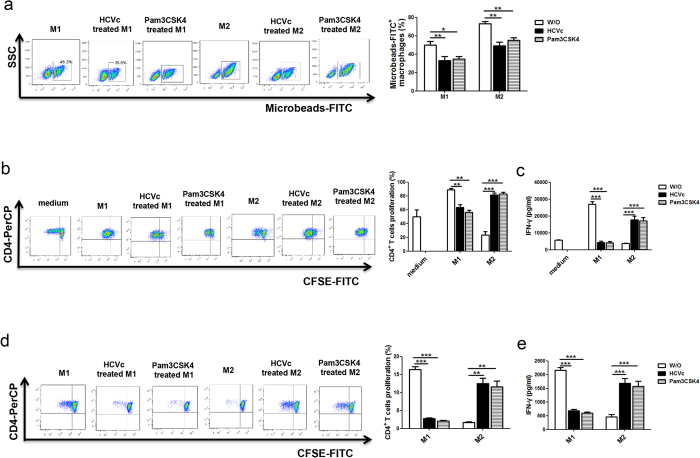
HCVc inhibits the functions of M1 and M2 macrophages. (**a**) Purified monocytes from healthy individuals were polarized to M1 and M2 macrophages in the presence or absence of HCVc or Pam3CSG4, FITC-microbeads were then added to culture for 1h. Phagocytosis was detected by flow cytometry. (**b,c**) Polarized macrophages in the presence or absence of HCVc or Pam3CSG4 were co-cultured with autologous activated CD4^+^ T cells by OKT3 and CD28. CD4^+^ T cells were stained with CFSE before co-cultured, CD4^+^ T cell proliferation were examined by flow cytometry, and IFN-γ secretion were assayed by ELISA. (**d,e**) Polarized macrophages in the presence or absence of HCVc or Pam3CSG4 were co-cultured with allogeneic CD4^+^ T cells. CD4^+^ T cells were stained with CFSE before co-cultured, CD4^+^ T cell proliferation were examined by flow cytometry, and IFN-γ secretion were assayed by ELISA. Shown is one of three representative experiments and statistical analysis. (*P < 0.05, **P < 0.01, ***P < 0.001).

Macrophages play a key role in immune surveillance by phagocytosis. We next examined whether HCVc affects phagocytosis of macrophages. Polarized macrophages in the presence or absence of HCVc and Pam3CSK4 were incubated with FITC-latex beads, and phagocytosis by macrophages was assessed by flow cytometry. The results were showed in Fig. 6a and Supplementary Fig. S4b. The ability of M2 macrophages to phagocytose FITC-microbeads was much stronger than M1 macrophages (72.9 ± 2.6 Vs 49.9 ± 4.1, *P* = 0.009), and HCVc significantly inhibited the phagocytosis of both M1 (49.9 ± 4.1 Vs 32.9 ± 4.5, *P* = 0.0097) and M2 (72.9 ± 2.6 Vs 48.9 ± 4.1, *P* = 0.0078) macrophages, as did Pam3CSK4 (M1: 49.9 ± 4.1 Vs 34.9 ± 2.5, *P* = 0.0357; M2: 72.9 ± 2.6 Vs 55.1 ± 2.8, *P* = 0.0092).

To further evaluate whether or not HCVc affects the immunomodulatory role of macrophages, polarized macrophages in the presence or absence of HCVc were co-cultured with autologous CD4^+^ T cells, activated by OKT3 and CD28. CD4^+^ T cells were stained with CFSE before being co-cultured, cell proliferation was examined by flow cytometry, and IFN-γ secretion was assayed by ELISA. The results are shown in Fig. 6b,c and Supplementary Fig. S4c. Compared with the medium control (without macrophages), M1 macrophages augmented CD4^+^ T cell proliferation (49.6 ± 10.4 Vs 88.8 ± 2.1, *P* = 0.0209) and IFN-γ secretion (5743.0 ± 223.7 Vs 26840.0 ± 1778.0, *P* < 0.0001), but M2 macrophages inhibited CD4^ + ^T cell proliferation (49.6 ± 10.4 Vs 23.6 ± 5.0, *P* = 0.0483) and IFN-γ secretion (5743.0 ± 223.7 Vs 3801.0 ± 170.0, *P* < 0.0001). HCVc-treated M1 macrophages down**-**regulated CD4^+^ T cell proliferation (88.8 ± 2.1 Vs 63.5 ± 3.9, *P* = 0.0047) and IFN-γ secretion (26840.0 ± 1778.0 Vs 4429.0 ± 50.0, *P* < 0.0001), compared with M1 macrophages. However, HCVc-treated M2 macrophages up-regulated CD4^+^ T cell proliferation (23.6 ± 5.0 Vs 81.2 ± 3.0, *P* = 0.0006) and IFN-γ secretion (3801.0 ± 170.0 Vs 17690.0 ± 2209.0, *P* < 0.0001), compared with M2 macrophages. Likewise, Pam3CSK4-treated M1 macrophages down-regulated CD4^+^ T cell proliferation (88.8 ± 2.1 Vs 56.1 ± 3.4, *P* = 0.0012) and IFN-γ secretion (26840.0 ± 1778.0 Vs 4223 ± 643.3, *P* < 0.0001), compared with M1 macrophages. Pam3CSK4-treated M2 macrophages up-regulated CD4^+^ T cell proliferation (23.6 ± 5.0 Vs 81.2 ± 3.0, *P* = 0.0006) and IFN-γ secretion (3801.0 ± 170.0 Vs 17190 ± 2035, *P* < 0.0001), compared with M2 macrophages.

To assess the antigen presenting function of macrophages, we then analyzed the capacity of polarized macrophages to stimulate the proliferation of allogeneic CD4^+^ T cells in mixed leukocyte reactions. Polarized macrophages were co-cultured with allogeneic CD4^+^ T cells. CD4^+^ T cells were stained with CFSE before co-cultured. The proliferation was examined by flow cytometry. IFN-γ secretion was assayed by ELISA. The results are shown in Fig. 6d,e. M1 macrophages induced much stronger allogeneic CD4^+^ T cell proliferation (16.4 ± 0.8 Vs 1.7 ± 0.2, *P* < 0.0001) and IFN-γ secretion (2159.0 ± 97.3 Vs 459.3 ± 85.8, *P* < 0.0001) than did M2 macrophages. HCVc-treated M1 macrophages down-regulated CD4^+^ T cell proliferation (16.4 ± 0.8 Vs 2.73 ± 0.26, *P* < 0.0001) and IFN-γ secretion (2156.0 ± 97.3 Vs 678.7 ± 58.9, *P* < 0.0001) compared with M1 macrophages. However, HCVc-treated M2 macrophages up-regulated CD4^+^ T cell proliferation (1.7 ± 0.2 Vs 12.5 ± 1.5, *P* = 0.0019) and IFN-γ secretion (459.3 ± 85.8 Vs 1685.0 ± 169.5, *P* < 0.0001) compared with M2 macrophages. Pam3CSK4-treated M1 macrophages down-regulated CD4^+^ T cell proliferation (16.4 ± 0.8 Vs 2.1 ± 0.1, *P* < 0.0001) and IFN-γ secretion (2156.0 ± 97.3 Vs 600.7 ± 32.78, *P* < 0.0001), compared with M1 macrophages. Pam3CSK4-treated M2 macrophages up-regulated CD4^+^ T cell proliferation (1.7 ± 0.2 Vs 11.54 ± 1.670, *P* = 0.0042) and IFN-γ secretion (459.3 ± 85.8 Vs 1574 ± 181.0, *P* = 0.0002), compared with M2 macrophages.

Thus, these results indicated that HCVc not only inhibits macrophage polarization, but also leads to dysregulation of the macrophages, including the phagocytic, antigen presenting, and immuno-modulatory function.

## Discussion

Monocytes and macrophages are key mediators of the immune response in the primary response to pathogens, tissue homeostasis, inflammation, resolution, and repair^27^. During HCV infection, monocytes and macrophages mediate an abnormal inflammatory response to influence the natural history of the infection^28^. Plasticity and functional polarization are hallmarks of macrophages that result in the phenotypic diversity of macrophage populations^29^. Deregulated macrophage polarization lead to pathogenesis during various disease conditions^30^. In the present study, we at first compared monocytes-derived macrophage polarization in HCV infected patients with healthy individuals, and found that both M1 and M2 macrophage polarization are impaired in chronic HCV infected (CHC) patients. In agreement with our findings, Fan C *et al.* recently reported that PBMCs from CHC patients receiving antiviral therapy (both responder and non-responder groups) were cultured with normal medium for 1 day prior to LPS/IFNγ M1 polarization, the polarized M1 macrophages had decreased TNF-α production^15^. These results indicate that HCV infection impairs normal M1 differentiation. New regimens of DAAs emerge with a cure rate of more than 90%, even in patients who failed on interferon therapy^31^. The NS5A inhibitor is one of DAAs known to disrupt multimeric arrays of dimers of NS5A and block the formation of the replication complex^32^. Given that DAAs do not directly stimulate cellular immunity, analysis of monocytes/macrophages during DAAs treatment with IFN-free regimens has the potential to provide new insight into innate immune responses during HCV infection. We further found that HCV viral clearance with DAAs partially restored the impaired macrophage polarization in HCV infection. One possible explanation is that suppression of HCV by DAAs releases the host immune responses from an active HCV suppression, augmenting the effectiveness of HCV therapies^33^. To our knowledge, this is the first description of macrophage polarization in chronic HCV infected patients treatment with DAAs. These results further confirm that HCV infection suppresses monocytes differentiation to both M1 and M2 macrophages.

It has been reported by our group and others that HCVc protein can activate TLR2 on human monocytes, macrophages, Kupffer cellsand regulatory T cells, which induces production of inflammatory cytokines by activating the MyD88-dependent TLR signaling pathway^17^,^23^,^34^,^35^. Therefore, we postulated that HCVc may engage with TLR2 on monocytes to regulate macrophage polarization. Peripheral monocytes from healthy individuals were differentiated to M1/M2 macrophages in the presence or absence of HCVc *in vitro*, and our data showed that HCVc inhibits monocyte polarization to M1 and M2 macrophages. Consistent with this, a previous study showed that HCVc and NS3/4A suppress M1 macrophage TNF-α and TfR1 expression and phagocytosis of HCVcc particles, which should be enhanced in the transformation of M1 to M2^15^. We further found that Pam3CSK4, a TLR2 ligation suppressed M1/M2 macrophage polarization as HCVc did, and blockade of TLR2 engagement with HCVc relieved the inhibition of macrophage polarization by HCVc. These results suggest that HCV manipulates macrophage polarization via HCVc engagement with TLR2 that is expressed on monocytes. Moreover, downstream signals like STAT1 phosphorylation are crucial for M1 macrophage polarization, and activation of STAT3 plays a pivotal role in the polarization of M2 macrophages, which are involved in anti-inflammation and wound healing^26^,^36^. Our result further showed that HCVc inhibits the phosphorylation of STAT1 in M1 macrophages and STAT3 in M2 macrophages. Similarly, it was reported that HCVc inhibits nuclear import of STAT1 in transiently transfected HuH-7 hepatoma cells, and inhibits both STAT1 and STAT2 in human osteosarcoma-derived cell lines that express HCVc^37^. However, suppression of STAT3 is not a universal action of HCVc, since the phosphorylation of STAT3 in macrophages polarized by PMA-stimulated THP-1 in the presence of exogenous HCVc was enhanced when co-cultured with L02 cells^12^.

Macrophages have great capacity for phagocytosis, which is the first step in antigen presentation^38^. We found that M2 macrophages displayed much stronger phagocytosis than M1 macrophages, and the phagocytosis of both M1 and M2 macrophages was down-regulated when treated with HCVc. Previous studies reported that transfection with *in vitro* transcribed HCVc DNA induced a significant decrease in phagocytosis of FITC-conjugated dextran in monocytes derived immature DCs^39^. These effects may have evolved as a mechanism by which HCV avoids phagocytosis by macrophages.

Macrophages play a crucial role in antigen presenting function and in the interaction between innate and adaptive immunity. M1 macrophages promote Th1 response and possess antiviral activity, while M2 macrophages are involved in promotion of the Th2 response, and of immune tolerance^40^. In chronic HCV infected patients, T cells are inefficient and defective in producing IFN-γ^41^. Here, we therefore asked whether the inhibition of macrophage polarization by HCVc could be partly responsible for this observation. We demonstrated that HCVc inhibits the allo-stimulatory capacity of M1 macrophages, but increases the allo-stimulatory capacity of M2 macrophages, which may favor an immune environment more supportive of viral replication. Other viruses may also manipulate macrophage biology to impair host defense. Macrophages isolated from rhesus macaque lymph nodes had the capacity to suppress CD4^+^ T cell proliferation and IFN-γ release after acute Simian Immunodeficiency Virus (SIV) infection^42^. It has been reported that THP-1 cells transfected with HCVc and treated with IFN-γ and LPS inhibit T cell proliferation and IFN-γ production by T cells derived from healthy donors^43^. These results also indicate that HCVc-treated M1 macrophages may contribute to the impairment of the T cell immune response during the early stage of chronic HCV infection.

In conclusion, this study revealed that monocyte polarization toward both M1 and M2 macrophages are impaired in patients with HCV infection through HCVc interaction with TLR2/STATs signaling, leading to dysfunction of both M1 and M2 macrophages. Kupffer cells, the tissue-resident macrophages in liver, display both pro-inflammatory and anti-inflammatory roles contributing to the immune response^44^,^45^. These effects could influence the behavior of both recruited M1 and M2 macrophages, and also resident Kupffer cells during HCV infection. Our study provides a new perspective on the mechanism of HCV persistent infection by an HCV encoded protein. We propose that HCV core protein blockade might be a novel therapeutic approach to this viral infection.

## Materials and Methods

### Ethics statement

All studies were conducted accordingto the experimental practices and standards that were approved by the Medical Ethics Committee of First Hospital of Jilin University (approval code: 2015–125), and informed consent was signed by patients enrolled in the study. In addition, all experiments were carried out in accordance with the approved guidelines and regulations.

### Samples

Seventeen chronic HCV-infected patients were enrolled in this study (Table 1). Venous bloods were withdrawn before treatment and after 7 days of administrated with direct-acting antiviral drug (NS5A inhibitor) for serum and peripheral blood mononuclear cells (PBMCs) collection. Buffy coats from seventeen healthy donors were provided by the Changchun Blood Center, and informed consent was provided according to the protocols of the Changchun Blood Center.

### HCVc and other regents

HCVc (amino acids2–192) and β-galactosidase was obtained from meridian life science Inc (Saco, USA). Human recombinant GM-CSF, M-CSF, IFN-γ, Lipopolysaccharide (LPS), IL-4, IL-13, Pam3CSK4 and anti-TLR2 antibody were purchased from R&D (NewYork, USA).

### Limulus amoebocyte assay for LPS contamination

Endotoxin contamination of Pam3CSK4 and HCVc was assessed using the QCL-1000 chromogenic end point assay (Cambrex, Cottonwood, AZ) and found to be less than 1 pg/mL.

### Cell isolation and purification

Peripheral blood mononuclear cells (PBMCs) were freshly isolated from peripheral blood of healthy individuals and patients with HCV infection by ficoll density gradient separation. Monocytes and CD4^+^ T cells were then purified by magnetic cell sorting with CD14 and CD4 microbeads (BD Bioscience, USA). The purity of the CD14^+^ cells and CD4^+^ T cells was equal or greater than 95% determined by flow cytometry.

### Cell cultures

Human monocytes were differentiated to M1/M2 macrophages as previously described^46^,^47^. In briefly, purified monocytes were differentiated into macrophages by GM-CSF (400IU/ml) or by M-CSF (50 ng/ml) respectively for 5 days in RPMI 1640 medium supplemented with 10% fetal calf serum (FCS), 100IU/ml penicillin and streptomycin at 37 °C with 5% CO_2_. For M1 macrophages polarization experiments, GM-CSF-induced macrophages were further exposed to fresh medium supplemented with 5% FCS and IFN-γ(20 ng/ml)/LPS (100 ng/ml) for additional 24 hours; For M2 macrophages polarization experiments, M-CSF-induced macrophages were further exposed to fresh medium supplemented with 5% FCS and IL-4 (25 ng/ml)/IL-13 (25 ng/ml) for additional 24 hours. For studying the effect of HCVc on macrophage polarization, HCVc (10 ug/ml), β-galactosidase (10 ug/ml) or Pam3CSK4 (1 ug/ml) were added to M1/M2 macrophages polarization. For blocking experiments, pretreated monocytes with anti-TLR2 antibody (0.15 ug/ml) for one hour were polarized to M1 and M2 macrophages in presence of HCVc.

### Monocytes siRNA transfection

Pure monocytes were transfected with siRNAs targeting TLR2 (Lafayette, CO) using Lipofectamine TM2000 transfection reagent (Invitrogen, Carlsbad, CA, USA) according to the manufacturer’sprotocol.

### Flow cytometry

Phenotypic analysises of M1 and M2 macrophages were performed on a flow cytometer (FACScan, BD Bioscience). M1 macrophages were identified using anti-human CD80-PE, anti-human CD86-FITC; M2 macrophage were identified by using anti-human CD163-PE and anti-human CD206-FITC. All the antibodies were obtained from BD Biosciences. The data acquired were analyzed with FlowJo (Treestar software, Ashland, OR, USA).

### Enzyme-linked immunosorbent assay (ELISA)

Concentrations of TNF-α, IL-10 and IFN-γ in cell culture supernatants were measured by ELISA according to the manufacturer’s instructions.

### Quantitative Real time- quantitative PCR (RT-qPCR)

Total cellular RNAs were isolated by Trizol (Invitrogen, Carlsbad, USA). Total RNAs were transcribed to cDNA by a reverse transcription kit (Transgen, China). Quantitative real-time PCR was conducted using the kit called Faststart Universal SYBR Green Master (ROX) (Roche, Germany). The primer sequence pairs were designed using NCBI online primer blast software. The primer sequence pairs for Inducible Nitric Oxide Synthase, iNOS were forward primer: 5′-CGTTGGATTTGGAGCAGAAGTG-3′ and reverse primer: 5′- CATGCAAAATCTCTCCACTGCC-3′. The primer sequence pairs for Arginase (Arg1) were forward primer: 5′-GGAATCTGCATGGGCAACCTGTGT-3′ and reverse primer: 5′-AGGGTCTACGTCTCGCAAGCCA-3′. Reactions were performed using 3 μl of cDNA in a 20 μl reaction volume and the following thermal cycles profile: 10 seconds for pre-denaturation at 94 °C, 5 seconds for denaturation at 94 °C, and 30 seconds for extension at 60 °C, for 40 cycles. Relative gene expression was determined by normalizing the expression of each target gene to GAPDH. All samples were assayed in triplicate and analyzed using the 2^−ΔCT^ method to assess relative fold change in iNOS and Arg1.

### Phagocytosis assay

Phagocytosis assay was executed as described in previous studies^48^,^49^. M1 and M2 macrophages polarizationin the presence or absence of HCVc were incubated with FITC-latex beads (0.05% of the stock concentration) in refreshed culture medium for 1 h at 37 °C with 5% CO_2_. Cells were then collected and measured by flow cytometry for phagocytic activity.

### CD4^+^ T cell proliferation

Pure monocyteswere polarized to M1 and M2 macrophages in the presence or absence of HCVc as mentioned above, the polarized macrophages were co-cultured with autologous and allogeneic CD4^+^ T cells as previously described^50^. Purified CD4^+^ T cells from autologous and allogeneic donors were stained with 10 μM CFSE (life technologies) according to the manufacturer’s recommended protocol. CFSE-labeled autologous and allogeneic CD4^+^ T cells were co-cultured with macrophages at macrophages/CD4^+^ T cells ratio = 1:4. For macrophages/autologous CD4^+^ T cells co-culture system, 10 μg/ml OKT3 (CD3 mAb; eBioscience, San Diego, CA. USA) were precoated to plates at the night before an experiment and kept in the refrigerator overnight, and anti-CD28 (5 μg/ml) antibody was then added to co-cultures as well. After 5 days, when clumps were visible, cells were collected and stained with anti-CD3-APC and anti-CD4-PerCP (BD Bioscience), and the proliferation of CD4^+^ T cells was analyzed by flow cytometry.

### Western blot

Polarized macrophages were lysed in ice-cold lysis buffer (RIPA buffer, protease inhibitor, phosphatase inhibitor cocktail 2 and 3, CST, USA) on ice. Protein quantification of cell lysates was completed using the Bradford (Bio-Rad, Hercules, CA) assay. Equal amounts of protein were separated by 10% SDS-PAGE and transferred to a Nitrocellulose membrane. The membranes were blocked with 5% non-fat milk in TBS-Tween solution (0.05% Tween 20 in Tris buffered saline) and then incubated overnight at 4 °C with primary antibodies. The blots were then washed with TBS-Tween solution and then incubated with the appropriate horseradish peroxidase-conjugated secondary antibodies (1:500) for 2 hours at room temperature, washed again with TBS-tween, and then developed with the enhanced chemiluminescence (ECL plus, Perkin Elmer, USA).

### Statistical analysis

All data were analyzed and found to be significant using the D’Agostino and Pearson omnibus normality test. Mean values were compared using either a paired t-test (two groups) or ANOVA (>two groups), followed by a Bonferroni correction for multiple comparisons test. *P* values < 0.05 were considered to be significant. All statistical tests were performed by Prism software (GraphPad, San Diego, CA, USA).

## Additional Information

**How to cite this article**: Zhang, Q. *et al.* HCV core protein inhibits polarization and activity of both M1 and M2 macrophages through the TLR2 signaling pathway. *Sci. Rep.* **6**, 36160; doi: 10.1038/srep36160 (2016).

**Publisher’s note:** Springer Nature remains neutral with regard to jurisdictional claims in published maps and institutional affiliations.

## Supplementary Material

Supplementary Information

## Figures and Tables

**Table 1:** Characteristics of study population.

Clinical data	Chronic HCV infected patients (n = 17)	Healthy controls (n = 17)	*P* value
Sex (male/female)	11/6	9/8	
Age (years)	44 ± 14.4	36.1 ± 6.5	0.177
ALT (U/L)	31 ± 6	21.7 ± 3.4	0.304
AST (U/L)	41.2 ± 10.6	15.1 ± 1.5	0.034*
Albumin (g/L)	43.8 ± 1	46.4 ± 1.1	0.079
TBIL (umol/L)	13 ± 4.9	9.9 ± 1.2	0.059
HCV RNA (IU/ml)	2.9 × 10^6^ ± 2.1 × 10^6^	<50	<0.0001***

Data are shown as average ± SD, *P* < 0.05 was considered statistically significant.

**Table 2:** Characteristics of chronic HCV infected patients administrated with DAAs.

Clinical data	Before treatment (n = 8)	After treatment (n = 8)	*P* value
Sex (male/female)	5/3	5/3	
Age (years)	43.2 ± 5.2	43.2 ± 5.2	
ALT (U/L)	65.4 ± 24.7	42.3 ± 23.9	0.520
AST (U/L)	47.3 ± 13.2	30.5 ± 10.7	0.352
Albumin (g/L)	38.5 ± 2.4	45.5 ± 2.2	0.065
TBIL (umol/L)	13.8 ± 1.1	11 ± 1.8	0.231
HCV RNA (IU/ml)	3.1 × 10^6^ ± 7.3 × 10^5^	3.6 × 10^3^ ± 1.1 × 10^3^	0.0009***
HCV core protein (fmol/ml)	2311 ± 848.5	202.9 ± 106.8	0.027*

Data are shown as average ± SD, *P* < 0.05 was considered statistically significant.
