# Supplementary material for: HCV core protein inhibits polarization and activity of both M1 and M2 macrophages through the TLR2 signaling pathway
Source: Sci Rep. 2016 Oct 27;6:36160. doi: 10.1038/srep36160 (PMC5082373; doi:10.1038/srep36160)
Supplement: Supplementary Information [file srep36160-s1.pdf]

Supplementary Information

HCV core protein inhibits polarization and activity of both M1 and M2 macrophages through the TLR2 signaling pathway

Qianqian Zhang[1,2,3], Yang Wang[1], Naicui Zhai[1], Hongxiao Song[1], Haijun Li[1], Yang Yang[1], Tianyang Li[1], Xiaolin Guo[2],Baorong Chi[2], Junqi Niu[2], Ian Nicholas Crispe[1,4], Lishan Su[1,5], Zhengkun Tu[1,2*]

Supplementary fig S1

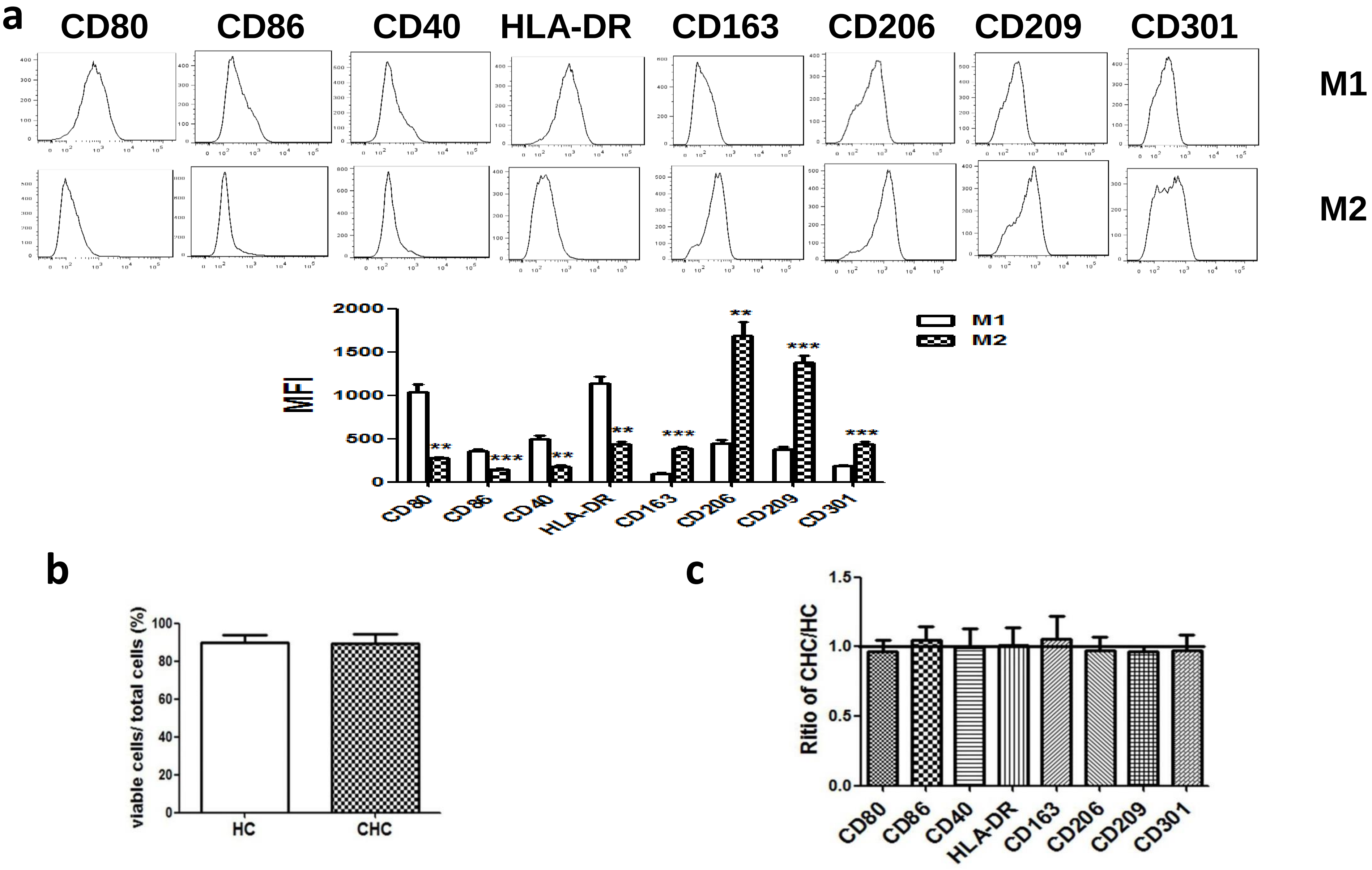

Supplementary fig S1: a, Phenotypic comparison of M1 and M2 macrophages from healthy donors. c, the viability of monocytes from healthy donors and chronic hepatitis C patients (CHC). d, Phenotypic comparison of monocytes from healthy donors and chronic hepatitis C patients (CHC).

Supplementary fig S2

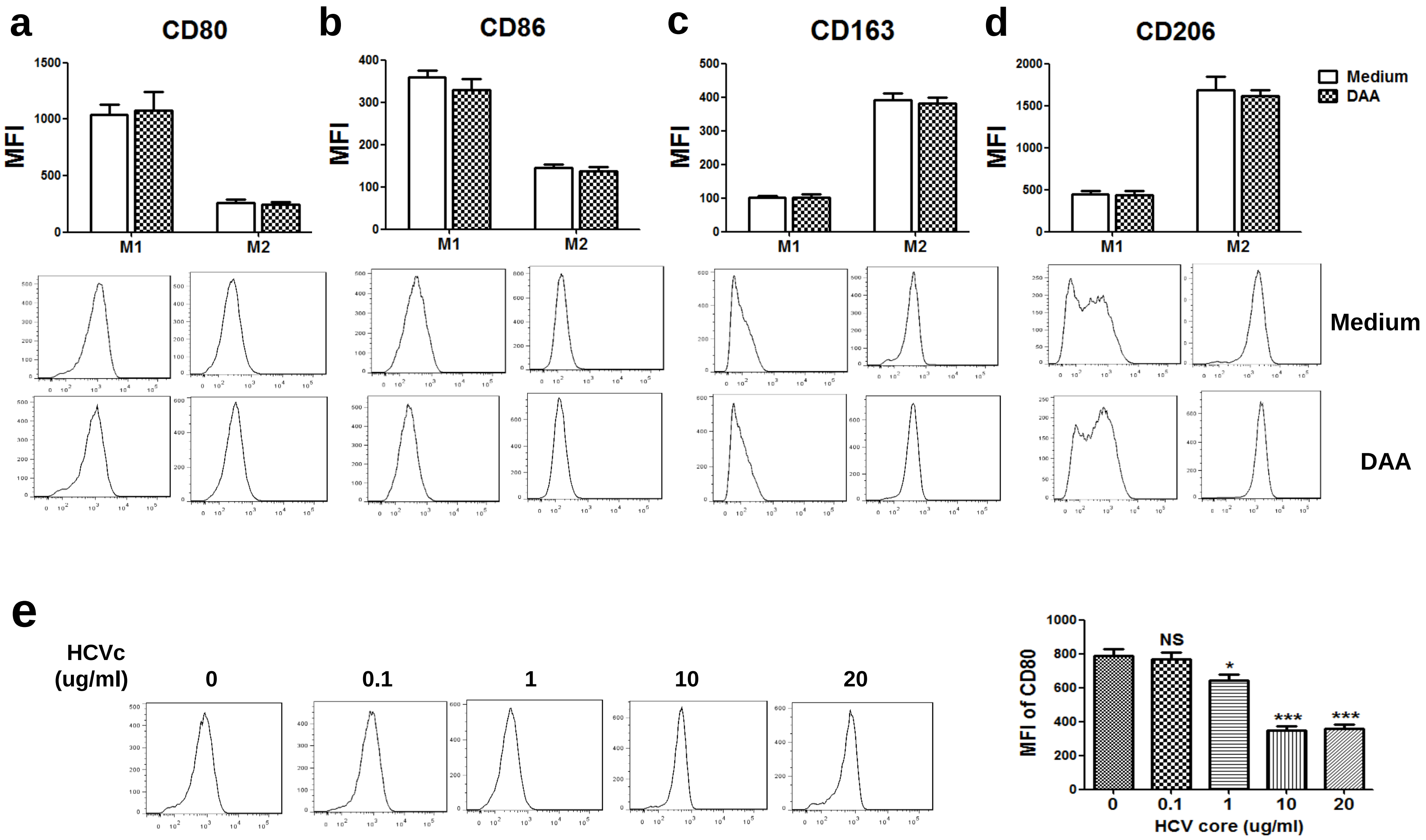

Supplementary fig S2: a-d, DAA does not suppress monocyte differentiation. Monocytes from healthy donors were differentiated to M1 and M2 macrophages in the presence or absence of DAA. e, HCVc inhibits expression of CD80 on M1 macrophages. Monocytes from healty donors were differentiated to M1macrophages in the presence of HCVc at concentrations ranging from 0 to 20ug/ml.

Supplementary fig S3

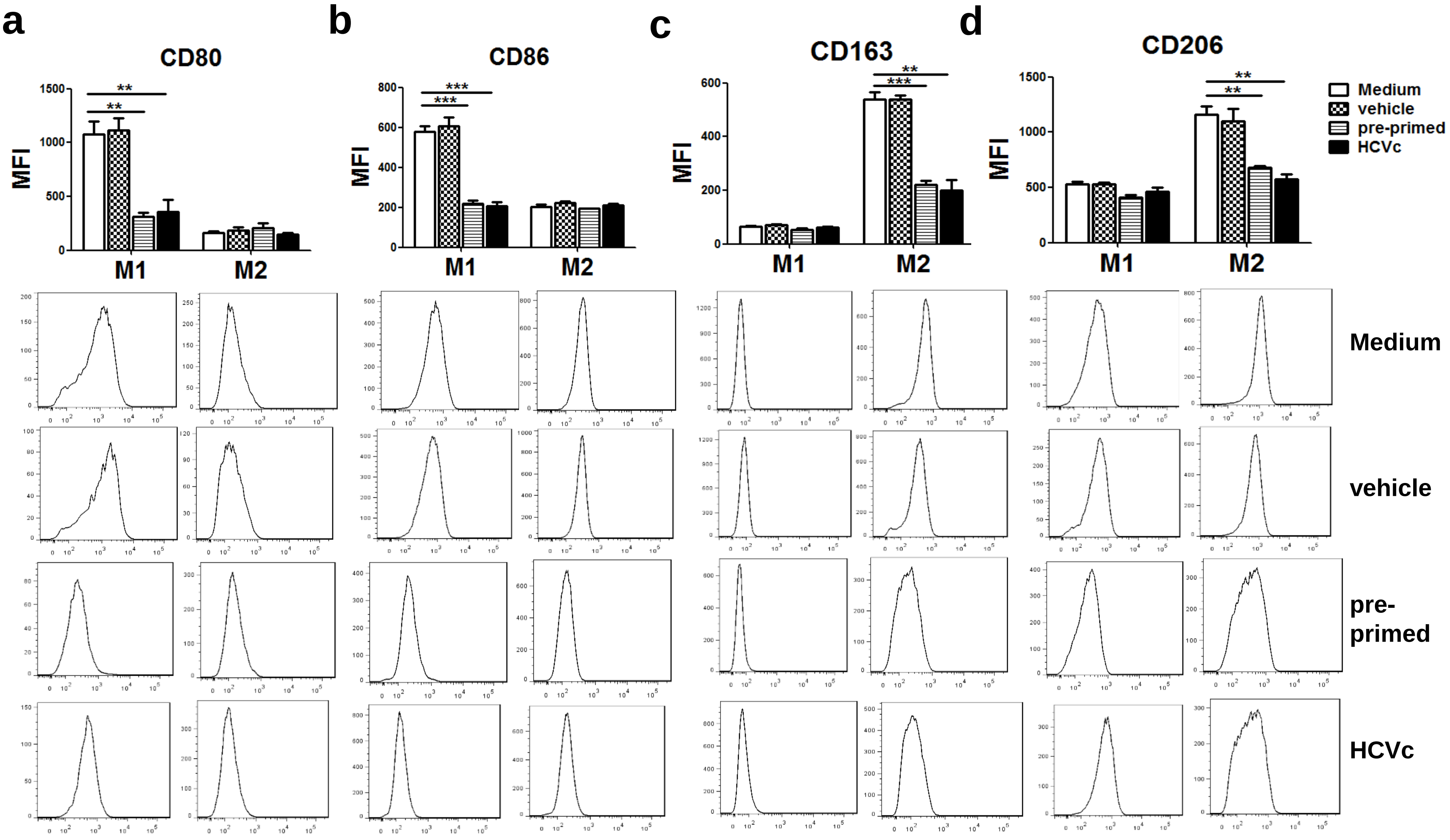

Supplementary fig S3: HCVc pretreatment inhibits the expression of CD80 and CD86 on M1 macrophages, CD163 and CD206 on M2macrophages as well as HCVc co-culture with monocytes during their differentiation, compared with the medium and vehicle. For HCVc pretreatment, monocytes from healthy donors were pre-treated with HCVc protein, and polarized M1 and M2 macrophages.

Supplementary fig S4

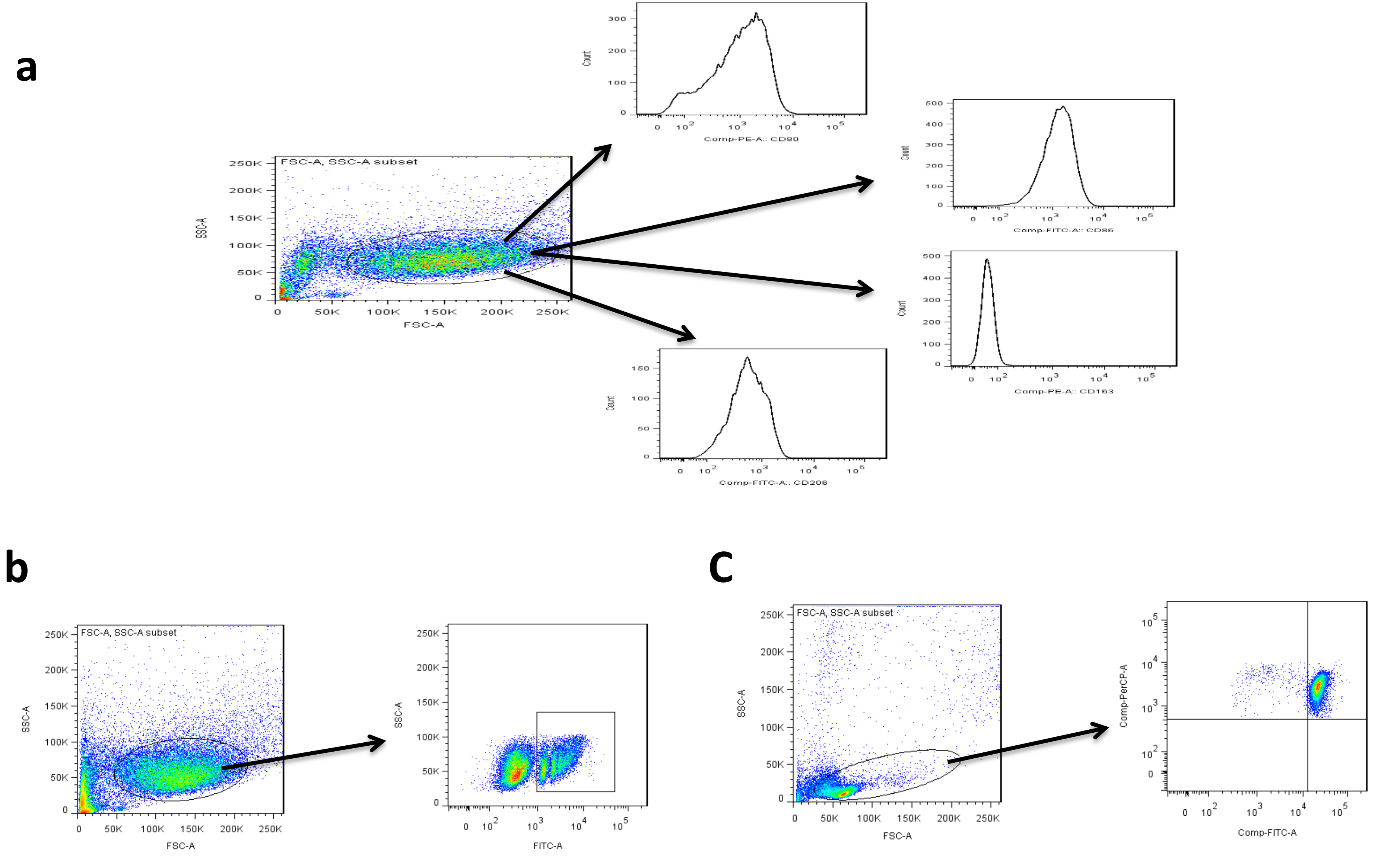

Supplementary fig S4: a, Macrophages gating strategy for CD80, CD86, CD163, and CD206 analysis. b, Macrophages gating strategy for phagocytosis analysis. c, $CD4^{+}$ T cells gating strategy for proliferation analysis.

Supplementary fig S5

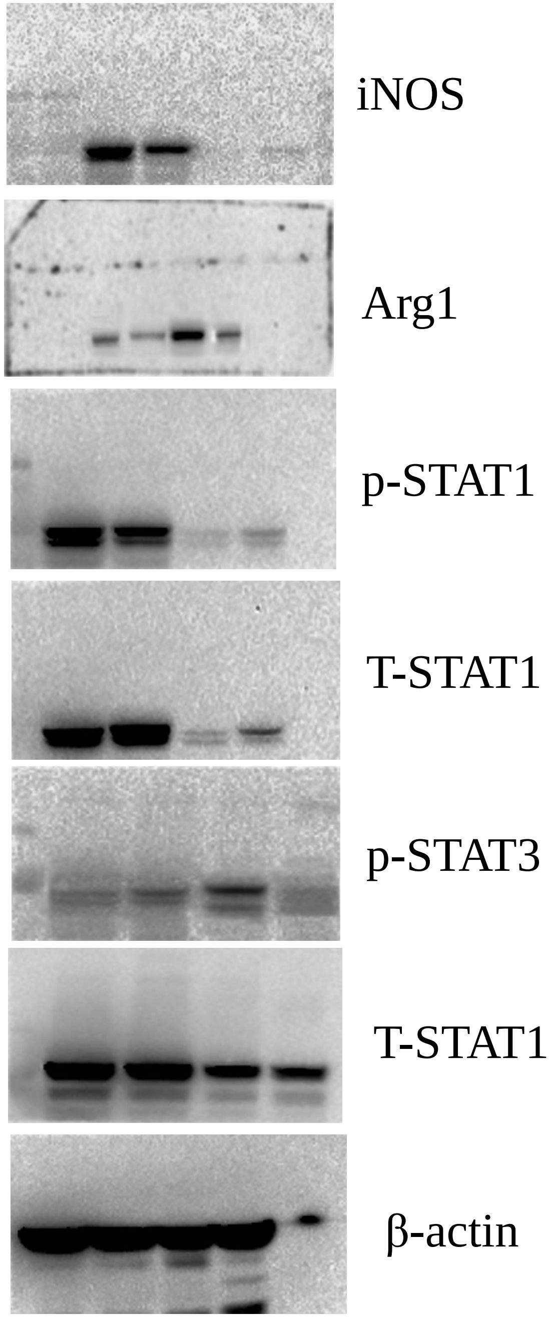

Supplementary fig S5: The original, full blot images.
